# Assessment of consent models as an ethical consideration in the conduct of prehospital ambulance randomised controlled clinical trials: a systematic review

**DOI:** 10.1186/s12874-017-0423-4

**Published:** 2017-09-16

**Authors:** Stephanie Armstrong, Adele Langlois, Despina Laparidou, Mark Dixon, Jason P. Appleton, Philip M. Bath, Helen Snooks, A. Niroshan Siriwardena

**Affiliations:** 1 Community and Health Research Unit, College of Social Science, University of Lincoln, Brayford Pool, Lincoln, LN6 7TS UK; 2 School of Social and Political Sciences, College of Social Science, University of Lincoln, Brayford Pool, Lincoln, LN6 7TS UK; 3 Stroke Trials Unit, Division of Clinical Neuroscience, University of Nottingham, Nottingham, UK; 4 Stroke Unit, Nottingham University Hospitals NHS Trust, Nottingham, UK; 5 Medical School, Grove Building, Swansea University, Singleton Park, Swansea, UK

**Keywords:** Ethics, Consent, Ambulance, Prehospital, Clinical trials

## Abstract

**Background:**

We sought to understand the main ethical considerations when conducting clinical trials in the prehospital ambulance based setting.

**Methods:**

A systematic review of the literature on randomised controlled trials in ambulance settings was undertaken. A search of eight databases identified published studies involving recruitment of ambulance service users. Four independent authors undertook abstract and full-text reviews to determine eligibility and extract relevant data. The data extraction concentrated on ethical considerations, with any discussion of ethics being included for further analysis. The resultant data were combined to form a narrative synthesis.

**Results:**

In all, 56 papers were identified as meeting the inclusion criteria. Issues relating to consent were the most significant theme identified. Type of consent differed depending on the condition or intervention being studied. The country in which the research took place did not appear to influence the type of consent, apart from the USA where exception from consent appeared to be most commonly used. A wide range of terms were used to describe consent.

**Conclusions:**

Consent was the main ethical consideration in published ambulance based research. A range of consent models were used ranging from informed consent to exception from consent (waiver of consent). Many studies cited international guidelines as informing their choice of consent model but diverse and sometimes confused terms were used to describe these models. This suggests that standardisation of consent models and the terminology used to describe them is warranted.

**Electronic supplementary material:**

The online version of this article (10.1186/s12874-017-0423-4) contains supplementary material, which is available to authorized users.

## Background

Prehospital or ambulance based research is a relatively new but rapidly developing field. A lack of randomised controlled trials (RCTs) in the prehospital setting has meant that most prehospital care relies on extrapolation from other sources. These are most often in-hospital trials that may face similar pressures such as timeliness of intervention or distress, but differ in other respects such as lack of access to resources or trained personnel and uncertainty of diagnosis [1].

In addition, extrapolation of evidence from in-hospital settings is not always valid since interventions that may be seen as the gold standard in hospital may not be feasible or of benefit in patients’ homes or during transport to hospital [2]. It has been argued that prehospital care itself is unique and therefore is likely to give rise to a different set of ethical considerations compared to research in other clinical care settings [3].

Ethical considerations in any research setting are complex and must account for a range of factors often based on risk/benefit analysis [4]. These ethical considerations are based on four basic principles, namely autonomy, non-maleficence, beneficence and justice [5]. In other words, people should be informed and free to choose whether to participate or not, the research should be potentially beneficial or in the participants’ ‘best interests’, risk of potential harm should be minimised, and studies should be equitable in recruitment and treatment of participants. The nature of a prehospital emergency situation may mean that the patient is not competent to make decisions about their best interest. The ability to achieve informed consent becomes problematic in light of the time pressures of emergency situations and the nature of the presenting conditions [3]. Researchers currently use a variety of models of consent to meet the requirements of legislation and to reflect the nature of the trial, which can lead to inconsistencies in the use of language and application of regulations.

The increasing use of paramedics in research, in particular in the randomisation and consent of patients to trials, highlights the need to review best practice in this area. In an interview study assessing ambulance based research, 92% of paramedics felt that research was important, yet only 35% actively took part [1]. Paramedics that did not participate identified barriers to research including the fear that randomisation and consent processes would lead to delays in treatment [1]. Therefore, it is important to understand the issues around gaining consent for research in the prehospital ambulance setting and to identify ways in which this can be achieved in practice.

In this study we sought to understand current ethical practices in prehospital ambulance trials. The aim of the review was to determine the range of approaches taken regarding consent models and to compare these approaches based on factors such as condition being studied and intervention used.

## Methods

The systematic review protocol was registered with PROSPERO (CRD42016038087). The protocol was designed using the Preferred Reporting Items for Systematics reviews and Meta-Analysis Protocols checklist (PRISMA) where possible, although due to the nature of the review not all of the checklist items were applicable [6]. We searched the following databases: MEDLINE, CINAHL, AMED, EBSCOhost, Science Direct, PsycARTICLES, PsycINFO and the Emergency Medicine Journal using these search terms in the title, abstract or key words: “emergency medical service*”, “ambulance”, “prehospital” and “emergency” with the additional key words: “intervention*”, “ethic*”, “procedure”, “ethic* approval”, “consent”, “confidentiality”, “trial”, “protocol”, and “randomised controlled trial”.

Studies were limited to published or ongoing randomised controlled trials of interventions involving ambulance services. More specifically, for studies to be considered eligible they had to have been conducted with ambulance service (or equivalent) users, including either adults or children (‘participants’); use any type of medical intervention involving ambulance service users (‘intervention’); explore health outcomes and provide information on relevant ethics procedures, such as consent and confidentiality (‘outcomes’); and be RCTs (‘study design’).

To ensure relevance to current practice and healthcare settings in developed countries we included studies conducted in the UK, Europe, North America, Australia and New Zealand between the years 2000 and 2016. Due to budget constraints papers were limited to those written in English. Studies were excluded if they did not involve ambulance service users or if they were case studies or guidance documents.

**Fig. 1:**
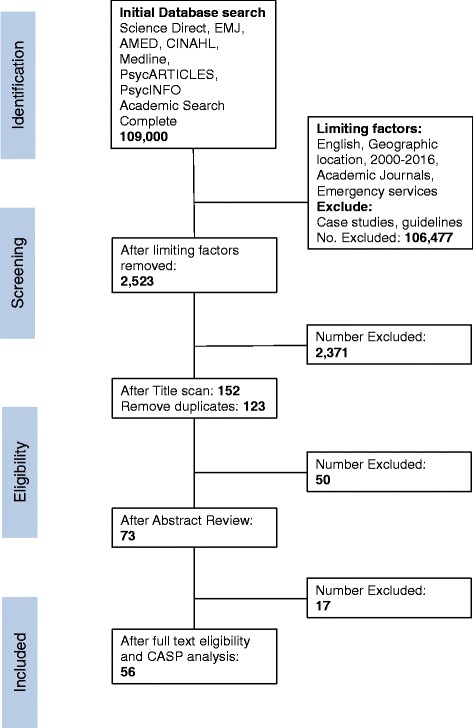
PRISMA flow chart

A review of titles then identified those most likely to be relevant and removed duplicates. Critical Appraisal Skills Programme (CASP) analysis was performed on the remaining papers to assess risk of bias and study quality [7]. CASP analysis uses a series of standard questions to determine if papers fulfil the requirements of the overall review question and in this case did not result in any papers being excluded [7]. Finally, a more detailed review of the abstracts was undertaken by 3 independent reviewers (DL, MD and JPA) resulting in 56 papers being put forward for full text review and data extraction. The PRISMA flowchart (Fig. 1) illustrates this process.

Data extraction was undertaken by four reviewers using the following headings: study design (RCT or cluster RCT), country, condition (disease or injury), intervention, blinding, participant details (age, gender and ethnicity), control group, inclusion criteria, exclusion criteria and general ethics (including any discussion of ethics from all sections of the paper). One reviewer (SA) extracted data from all 56 papers, while the remaining reviewers (DL, MD and JPA) completed extraction on 18 or 19 papers each. The results of the data extraction were combined to ensure that all relevant information was captured.

Once data extraction was completed, narrative analysis was used to synthesise the data, determine any gaps or inconsistencies and to highlight any issues to be addressed. The data were analysed using MS Excel for fields containing empirical or short format data. For fields containing large passages of text namely inclusion and exclusion criteria, and general ethics information the text was combined and analysed to identify themes supported by NVivo 10. These themes were then assessed to determine whether any relationships could be identified between them.

## Results

**Fig. 2:**
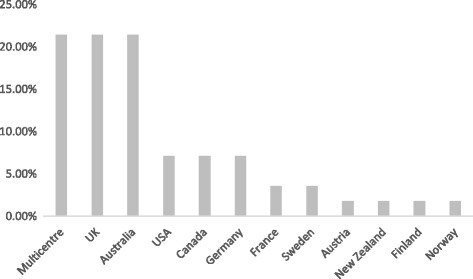
Occurrence of each country as a percentage of the total (*N* = 56)

**Fig. 3:**
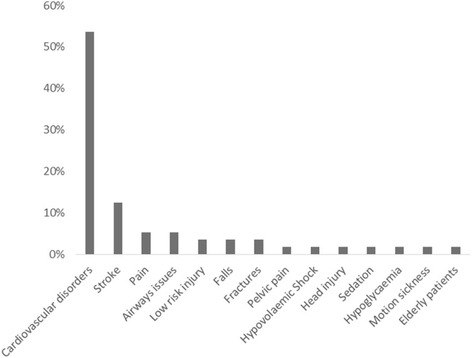
Occurrence of each condition as a percentage of the total (*N* = 56)

**Fig. 4:**
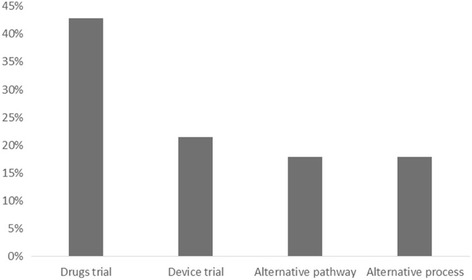
Occurrence of each intervention as a percentage of the total (*N* = 56)

The main characteristics of the 56 papers included in the review (Additional file 1), namely country, condition and intervention, are summarised in Figs. 2, 3 and 4. English-speaking countries were the most represented in the papers but this was not surprising given the language limitations of the review. There were also a high number of multicentre papers (12, 21.4%), where research had been undertaken in more than one country. Some studies involved neighbouring countries (for example USA and Canada or Germany and Netherlands) whilst others included a wide range of centres; one study had 12 research centres across Europe, North America, Australia and North Africa [8] (Fig. 2). For the multicentre papers the individual countries are listed in full in Additional file 1.

The studies analysed were mostly emergency situations, with cardiovascular conditions (28 studies, 49.4%) such as cardiac arrest occurring most frequently, followed by stroke (7 studies, 12.5%) and lower risk injuries and illnesses (Fig. 3). Interventions fell broadly into four categories (Fig. 4). Firstly, drug trials (24 studies, 42.9%) usually involved the early administration of drugs normally given on arrival at hospital, comparisons of two routinely used drugs or novel agents, for example 100% oxygen to prevent motion sickness during transport. Secondly, device trials (12 studies, 21.4%) included comparison of different airways devices or the use of automated chest compression units in comparison to manual chest compressions. Thirdly, alternative pathway trials (10 studies, 17.9%) sought to relieve the pressures on emergency departments (ED) by routing patients either to community care or directly to in hospital treatment, bypassing the ED. Finally, alternative process trials (10 studies, 17.9%) assessed the use of different procedures in the ambulance setting, for example the use of CPR prior to defibrillation.

The data pertaining to ethical considerations comprised larger pieces of text and so were coded and analysed using NVivo 10. We classified the coded data into three groups (‘consent issues’, ‘approval issues’ and ‘other’), which highlighted the main themes for further analysis.

All of the papers reviewed discussed methods used to gain consent to some degree. Approval issues included discussion of ethics committee approvals and in some cases the regulations that were adhered to as part of the ethics approval, although reporting of this was inconsistent and not all papers included this information (34 of 56). The ‘other’ group contained the smallest number of papers (11 of 56) and covered various topics. For example, one paper discussed the refusal of a study site to participate, another the impact of media reports on the project and a third highlighted cost implications. Consent appeared to be the paramount consideration in the reporting of prehospital research and therefore this area was subject to further analysis.

In order to produce a narrative analysis of the data, comparisons were made between the type of consent used and the country in which the study took place, the condition under investigation and the intervention used. The type of consent will have been influenced by the legislation and regulations of the country in question, although several papers did refer to international guidelines such as the Declaration of Helsinki or the Good Clinical Practice (GCP) guidelines [9, 10](Table 1).

**Table 1:** A comparison of type of consent obtained and the country where the research took place. (N = number of trials. Note some trials used more than one type of consent)

Type of Consent	Multicentre (*N* = 12)	UK (*N* = 12)	Australia (*N* = 12)	USA (*N* = 4)	Canada (*N* = 4)	Germany (*N* = 4)	France (*N* = 2)	Sweden (*N* = 2)	Austria (*N* = 1)	New Zealand (*N* = 1)	Finland (*N* = 1)	Norway (*N* = 1)	Total
Waived Consent	4	3	6	3	3	1	1	–	–	–	–	1	22
Informed Consent	7	2	2	–	1	3	1	2	–	1	–	–	19
Relative Proxy	1	4	1	–	1	–	–	2	–	1	1	–	11
Delayed Consent	1	1	3	–	–	2	1	1	–	–	–	–	9
Consent for Follow Up	1	2	1	–	–	–	1	–	–	–	–	–	5
Verbal Consent	–	2	1	–	–	–	1	–	–	–	–	–	4
Paramedic Proxy	–	2	–	–	–	–	–	–	–	–	–	–	2
Retrospective Consent	–	1	1	–	–	–	–	–	–	–	–	–	2
Paramedic Consented	–	–	–	1	–	–	–	–	–	–	–	–	1
Opt Out	–	1	–	–	–	–	–	–	–	–	–	–	1

Many of the studies used more than one method for gaining consent, which is reflected in Table 1. The widest range of consent types used was in the United Kingdom (UK). This may reflect the number of UK studies reviewed, but also that UK trials often use multiple consent models such as informed consent and a variety of proxy consent processes (relatives as proxies being the most common) [11]. (The term proxy consent in this paper refers to gaining either legal consent or opinion from a person other than the participant, sometimes referred to as surrogate consent.)

Informed consent was the most widely used form of consent across the different countries. In the USA exception from informed consent (waiver of consent) was most common and was used in all but one of the studies, in which paramedics consented to their own participation. In other countries where waiver of consent was used this was often coupled with some form of additional consent, such as consent for follow-up data collection and the opportunity for participants to withdraw their involvement [12–14]. Multicentre papers tended to use a single form of consent, either waiver of consent or informed consent, with only two of the twelve multicentre papers using more than one type of consent [15]. This suggests ethical regulations and in particular consent requirements are not barriers for international trials, and that the differences seen in UK trials could be due to other factors such as the conditions being studied. Analysis showed that the UK studies included four stroke trials, all of which used multiple consent models. The multicentre trials predominately involved cardiovascular disorders such as myocardial infarction or cardiac arrest, participants in these trials may have been more likely to uniformly lack capacity therefore simplifying the consent process.

A comparison of type of consent with the condition being studied showed that where patients lacked capacity for example due to cardiac arrest, waiver of consent was the most commonly used model (Table 2). In patients who survived the initial illness, additional consent for follow-up data collection was often used [14, 16, 17]. In these cases, there was also commonly provision for relative proxy consent or delayed consent whereby patients were asked to complete a consent form when they recovered capacity [18–20]. Trials involving stroke patients tended to have the most varied consent models and usually included more than one type of consent, reflecting the varying severity of strokes, and potential loss of capacity, and the complex nature of the condition. This is reflected in Table 2 as, whilst there were only seven stroke trials reported, stroke accounted for 18 models of consent. The majority of trials where patients were likely to have capacity, even in an emergency situation, asked patients to provide written informed consent in the prehospital setting (usually in the ambulance). Several studies highlighted that even when a patient was conscious factors such as pain could influence their capacity to give informed consent [21, 22].

**Table 2:** Comparison of type of consent with the condition being studied (N = number of trials. Note some trials used more than one type of consent)

Type of Consent	Cardiac Arrest (*N* = 19)	STEMI (*N* = 9)	Stroke (*N* = 7)	Airways Issues (*N* = 3)	Pain (*N* = 3)	Other Heart Conditions (*N* = 2)	Low Risk Injury (*N* = 2)	Fractures (*N* = 2)	Falls (*N* = 2)	Pelvic Pain (*N* = 1)	Hypovolaemic Shock (*N* = 1)	Head Injury (*N* = 1)	Sedation(*N* = 1)	Hypoglycaemia (*N* = 1)	Motion Sickness (*N* = 1)	Elderly Patients (*N* = 1)	Total
Waived Consent	13	–	1	2	1	2	–	–	–	–	1	1	1	–	–	–	22
Informed Consent	–	7	5	–	1	–	1	2	–	1	–	–	–	–	1	1	19
Delayed Consent	4	2	1	–	–	–	–	–	–	–	–	–	–	1	–	–	8
Relative Proxy	3	1	4	1	–	–	–	1	–	–	–	–	–	–	–	1	11
Paramedic Proxy	–	–	2	–	–	–	–	–	–	–	–	–	–	–	–	–	2
Consent for Follow Up	5	–	3	–	1	1	–	–	–	–	–	–	–	–	–	–	10
Verbal Consent	–	–	2	–	1	–	1	1	–	–	–	–	–	–	–	–	5
Retrospective Consent	–	–	–	–	–	–	–	–	1	–	–	1	–	–	–	–	2
Paramedic Consented	–	–	–	1	–	–	–	–	–	–	–	–	–	–	–	–	1
Opt Out	–	–	–	–	–	–	–	–	1	–	–	–	–	–	–	–	1

Analysis of consent type compared with intervention found that most trials involving drug treatments required informed consent or some form of proxy consent (Table 3). Where waiver of consent was used this was often accompanied by consent for follow-up data usage in survivors [11]. Trials evaluating alternative pathways also tended to use written informed consent in the ambulance. Patients in these trials were more likely to have capacity and most would not have required transportation to hospital, and therefore informed consent was obtainable [23]. Two papers in this group used what was termed retrospective consent to describe a process whereby gaining consent was delayed until after the initial incident had passed [21, 24]. Device trials most frequently used waiver of consent, the devices used in these trials being mainly airway or automated chest compression devices [12, 25]. In both cases the illnesses necessitating the use of these devices would render the participants incapable of giving informed consent themselves.

**Table 3:** Comparison of type of consent with the type of intervention being studied. (N = number of trials. Note some trials used more than one type of consent)

Type Of Consent	Drug V Placebo (*N* = 24)	Alternative Pathway V Normal Care (*N* = 10)	Alternative Process V Normal Care (*N* = 10)	Device Trial V Placebo or Normal Care (*N* = 12)	Total
Waived Consent	8	2	3	9	22
Informed Consent	11	6	1	1	19
Delayed Consent	2	1	4	1	8
Relative Proxy	4	2	3	2	11
Paramedic Proxy	2	–	–	–	2
Consent For Follow Up	6	–	1	3	10
Verbal Consent	5	–	–	–	5
Retrospective Consent	–	2	–	–	2
Paramedic Consented	–	–	–	1	1
Opt Out	–	1	–	–	1

## Discussion

This review analysed 56 studies undertaken in the prehospital setting by paramedics (or equivalent) for a range of conditions and interventions. Consent was the paramount consideration when reporting ethical issues around clinical trials undertaken in the prehospital ambulance based setting. Analysis of the methods for obtaining consent showed relationships between the type of consent and both the condition and the intervention being assessed.

For conditions or interventions where participants were more likely to lack capacity, waiver of consent, proxy consent (usually from a relative) or delayed consent models were used. The review also analysed the type of consent in relation to the country in which the research took place. From the data, country did not appear to have a direct impact on the type of consent model used, with the exception of the United States (USA), where exception from consent (waiver of consent) was the model of choice. US Food and Drug Administration (FDA) regulation 21CFR50.24 clearly sets out the requirements for emergency research and the use of exception from consent would appear to be the accepted norm [26].

Many of the studies reviewed contained statements regarding ethical approvals, with several including statements regarding compliance with international guidance, in particular the Declaration of Helsinki or Good Clinical Practice (GCP) guidelines [9, 10]. Most of the studies from the USA mentioned compliance with the FDA 21CFR50.24 regulations, which provide criteria allowing exception from informed consent [26]. All of these regulations or guidelines set standards for obtaining consent in medical research in a hierarchy of preference where written informed consent from the participant is the favoured method, followed by consent from a personal or legal representative (proxy consent).

Most legislation includes emergency provisions allowing participants to be recruited to the study provided they (or their representative) are informed at the earliest opportunity and they are given the option to withdraw from the study. Whilst several trials did indicate the number of participants withdrawing from the study, most did not, nor did they indicate that participants were given this option [12, 27, 28]. This is an area where reporting of ethical considerations in clinical trials could be more explicit.

Terminology relating to consent is an area that needs greater consistency, particularly where emergency exception or waiver of consent is used. Whilst many papers did use the terms exception from or waiver of consent, in line with guidance documents, several used other terms such as delayed or retrospective consent [21, 23, 24, 29]. The term delayed consent does not appear in any of the guidance documents, but in effect describes the emergency exception from consent model, whereby consent must be sought as soon as possible either from the participant or their representative. Retrospective consent in these studies was used in the same way as a means of describing the process of emergency exception and later consent, but the use of this term could be problematic. Previously the term ‘retrospective consent’ has been referred to in psychology research as consent that is sought after the intervention in order to correct a deliberate deception on the part of the researcher. In psychology research deliberate deception may be necessary for the integrity of the research and the term retrospective consent is used in those circumstances where the researcher later informs the participant of the true nature of the research and gains consent to replace any consent given before the intervention [30]. Since deliberate misrepresentation would not occur in clinical trials in the prehospital environment, the use of the term retrospective consent may be confusing.

The use of terminology was not specific to the country in which the research took place and terms for type of consent were used interchangeably across all countries, conditions and interventions. This supports the need for standardisation of terminology for consent in research and subsequent publications.

Further consideration should also be given to the nature of informed consent. The Declaration of Helsinki and the GCP guidelines state that exception from consent should only be used where there is a medical emergency where timeliness of the intervention means that it cannot be delayed in order to gain consent from a legal representative [9, 10]. In general this is limited to conditions such as cardiac arrest or stroke where the outcome for the patient could be affected by a delay in treatment. This means that, when a patient has called an ambulance in a situation not technically classed as a medical emergency, any research intervention will require consent from the patient or a surrogate. Several papers discussed the issue of gaining consent in the prehospital setting when a participant has called an ambulance in a situation not classed as a medical emergency [21, 22]. Situations that necessitate calling an ambulance are by their nature stressful, not only to potential participants but also to their relatives, who might act as their representatives. This suggests that consent gained under these circumstances may not be a true reflection of the participant’s wishes if they or their surrogate had more time and a less stressful situation in which to consider taking part.

Research in the prehospital setting may therefore require a different approach to consent, whether this is through a broadening of exception from consent to include situations not classed as medical emergencies or through a different approach entirely, such as assent. The Declaration of Helsinki does briefly discuss the use of assent for individuals who cannot give informed consent for inclusion in research but do have the capacity to agree to treatment. This would then be coupled with informed consent from the participant or from their legal representative as soon as possible. This is an aspect that needs further exploration, discussion and agreement by the wider research community.

Although this review has focussed on consent because of the nature of the review question and process, there are a range of other ethical issues raised by prehospital trials. Aspects such as patient perceptions and practitioner views of prehospital research have begun to be explored in previous studies [31]; these and other ethical concerns of ambulance trials such as the balance of risk and benefits or equity of participation should be addressed in future studies in order to guide future research design [32].

### Limitations

The search strategy was comprehensive, however we acknowledge that because it was limited to available databases and papers published in the English language (due to time and budget constraints), all relevant papers may not have been identified. Our intention with this paper was to identify ethical considerations in clinical trials undertaken in ambulance settings through the review of published RCT papers and as such the analysis was limited to only those ethical considerations discussed in the resultant papers. However, it may be that ethical issues occurred during these trials that were not reported in the resultant publications. Moreover, it may be that additional ethical considerations (beyond consent) are reported elsewhere, for example in discussion or case study articles that focus on prehospital ambulance based research more generally.

## Conclusion

Prehospital ambulance research is a developing field that aims to expand the evidence base with the aims of improving outcomes for patients and implementing cost-effective approaches for healthcare provision. Prehospital care is unique due to the environmental and time pressures of delivering clinical interventions in this setting, which will impact on ethical considerations. This systematic review of the literature found that the ability to obtain consent was the overarching ethical consideration. The stressful nature of ambulance calls leads to questions regarding the ability to obtain written informed consent. Whether alternative methods such as wider use of emergency exception from consent, or assent coupled with consent to include follow-up data collection should be standard for this type of research is unclear. The use of terminology to describe consent models was also highly varied and standardisation of terminology would be beneficial for clarity of consent (for both participants and investigators) and ethical considerations in prehospital clinical trials.

## Additional file

Additional file 1: Full reference list and basic summary of data collected from the papers reviewed. (DOCX 36 kb)

## Abbreviations

CASP: Critical Appraisal Skills Programme
ED: Emergency Department
GCP: Good Clinical Practice
PRISMA: Preferred Reporting Items for Systematics reviews and Meta-Analysis Protocols
RCT: randomised controlled trials
