# Supplementary material for: Assessment of consent models as an ethical consideration in the conduct of prehospital ambulance randomised controlled clinical trials: a systematic review
Source: BMC Med Res Methodol. 2017 Sep 16;17:142. doi: 10.1186/s12874-017-0423-4 (PMC5603026; doi:10.1186/s12874-017-0423-4)
Supplement: Additional file 1: — Full reference list and basic summary of data collected from the papers reviewed. (DOCX 36 kb) [file 12874_2017_423_MOESM1_ESM.docx]

| **Author(s)** | **Country** | **Condition** | **Intervention** | **Model of consent** |
| --- | --- | --- | --- | --- |
| Ankolekar *et al.* (2013) | UK | Stroke | GTN patch/placebo | Written informed consent for patient with capacity, relative proxy consent or paramedic proxy consent for those lacking capacity. Consent to continue in the trial sought once patient recovered capacity in hospital. |
| Ankolekar *et al.* (2012) | UK | Stroke | GTN patch/placebo | Written informed consent for patient with capacity, relative proxy consent or paramedic proxy consent for those lacking capacity. Consent to continue in the trial sought once patient recovered capacity in hospital. |
| Arendts *et al*. (2011) | Australia | Low risk injury | Transfer to ED/treat at home | Written informed consent on scene |
| Baker *et al*. (2008) | Australia | Cardiac arrest | CPR before defibrillation/ normal care | Waiver of consent |
| Barker *et al.* (2006) | Austria, Italy, Hungary | Pelvic pain | TENS/placebo | Written informed consent prior to intervention |
| Benger *et al*. (2016) | UK | Cardiac arrest | i-gel supraglottic airway insertion/laryngeal mask airway supreme | Paramedics consented to take part in trial. Patients enrolled under waiver of consent, consent for follow up inclusion sought once they regained capacity. |
| Bernard *et al.* (2012) | Australia | Cardiac arrest | Prehospital therapeutic hypothermia/in-hospital therapeutic hypothermia | Waiver of consent |
| Brasel *et al*. (2008) | USA, Canada | Hypovolemic shock | Saline solution/normal care | Waiver of consent |
| Breil *et al*. (2012) | Germany | Cardiac arrest | Different types of saline solution | Delayed consent sought from patients who survived resuscitation or their relatives if they did not recover capacity. |
| Brown *et al*. (2015) | USA, Canada | Cardiac arrest | Continuous chest compression/ interrupted chest compression | Waiver of consent |
| Dixon *et al.* (2009) | UK | Minor injury | Paramedic practitioner/normal care | Waiver of consent with consent for follow up |
| Ducasse *et al.* (2013) | France | Traumatic injury | 30 mins nitrous oxide and air/15 mins nitrous oxide and air | Initial verbal consent followed by full informed consent in hospital |
| Ebinger *et al*. (2014) | Germany | Stroke | Stroke emergency mobile unit (STEMO)/normal care | Delayed consent sought once patients regained capacity coupled with informed consent to use data. |
| Ezekowitz *et al*. (2014) | Canada | Myocardial infarction | BNP and troponin assay/normal care | Full informed consent prior to intervention. |
| Frascone *et al*. (2011) | USA | Airways compromised | Supraglottic airway/ET tube | Consent sought from paramedics not patients |
| Garner *et al*. (2015) | Australia | Head Injury | Physician intervention/paramedic only | Waiver of consent with retrospective consent for follow up |
| Hassan *et al*. (2002) | UK | Ventricular fibrillation | Magnesium sulphate/placebo | Waiver of consent |
| Heestermans *et al*. (2011) | Netherlands, Germany, Belgium | STEMI | Tirofiban/placebo | Full informed consent |
| Heestermans *et al*. (2010) | Netherlands, Germany, Belgium | STEMI | Tirofiban/placebo | Full informed consent |
| Jabre *et al.* (2011) | France | Airways compromised | Single use metal laryngoscope/standard metal laryngoscope | Waiver of consent |
| Jacobs *et al*. (2011) | Australia | Cardiac arrest | Adrenaline/placebo | Waiver of consent |
| Jacoby *et al*. (2006) | USA | Sedation for intubation | Etomidate/midazolam | Waiver of consent |
| Jennings *et al*. (2012) | Australia | Pain | Ketamine and morphine/morphine alone | Waiver of consent |
| Jennings *et al*. (2014) | Australia | Long-term pain | Ketamine and morphine/morphine alone | Consent for follow up |
| Kämäräinen *et al*. (2009) | Finland | Cardiac arrest | Therapeutic hypothermia with cooled Ringers acetate/normal care | Relative proxy consent |
| Kim *et al*. (2014) | USA | Cardiac arrest | Therapeutic hypothermia with cooled saline/normal care | Waiver of consent during emergency incident with additional consent for follow up |
| Kober *et al*. (2002) | Austria | Motion sickness | 100% oxygen/air | Full informed consent |
| Kovoor *et al*. (2005) | Australia | Cardiac arrest | Sotalol/Lignocaine | Waiver of consent |
| Larsson *et al*. (2016) | Sweden | Hip fracture | Fast track to radiology/normal delivery to accident and emergency | Full informed consent or relative proxy consent where patient lacks capacity |
| Lerner *et al*. (2011) | USA | Cardiac arrest | Autopulse CPR/manual CPR | Waiver of consent |
| McRae *et al*. (2015) | Australia | Femoral fracture | Femoral nerve blocks/normal care | Initial verbal consent with informed consent sought once in hospital |
| Middleton *et al*. (2014) | Australia | Cardiac arrest | i-gel supraglottic airway insertion/ Portex soft seal LM | Waiver of consent |
| Montalescot *et al*. (2013) | France, Germany, Netherlands, United Kingdom, Austria, Denmark, Sweden, Italy, Spain, Canada, Australia, Algeria | STEMI | Tricagrelor prehospital and in hospital placebo/Prehospital placebo and in hospital tricagrelor | Written informed consent in the ambulance |
| Moore & Wollard (2005) | UK | Hypoglycaemia | 10% dextrose/normal care 50% dextrose | Delayed consent once patients recovered capacity |
| Morrison *et al*. (2005) | Canada | Cardiac arrest | Bi-phasic defibrillation/mono-phasic defibrillation | Waiver of consent |
| Morrison *et al*. (2008) | Canada | Haemodynamically unstable bradycardia | Transcutaneous cardiac pacing with dopamine/normal care | Waiver of consent along with additional consent for follow up |
| Olasveengen *et al*. (2012) | Norway | Cardiac arrest | Adrenaline/no adrenaline | Waiver of consent along with additional consent for follow up |
| Paradis *et al*. (2010) | USA/Canada | Cardiac arrest | Autopulse CPR/manual CPR | Waiver of consent |
| Perkins *et al*. (2015) | UK | Cardiac arrest | LUCAS-2 CPR/manual CPR | Waiver of consent along with additional consent for follow up |
| Perkins *et al.* (2010) | UK | Cardiac arrest | LUCAS-2 CPR/manual CPR | Waiver of consent along with additional consent for follow up |
| Rubertsson *et al*. (2014) | Netherlands, Sweden, UK | Cardiac arrest | LUCAS-2 CPR/manual CPR | Delayed consent with relative proxy consent for patients that do not regain capacity |
| Shaw *et al*. (2011) | UK | Stroke | Lisinopril/normal care | Verbal consent or relative proxy consent where patient lacks capacity |
| Shaw *et al*. (2014) | UK | Stroke | Lisinopril/normal care | Verbal consent initially with relative proxy consent where patients lacks capacity along with additional consent for follow up |
| Snooks *et al*. (2012) | UK | Falls | Healthcare pathway/normal care | Initial waiver of consent with retrospective consent |
| Snooks *et al*. (2010) | UK | Falls | Software to aid paramedic decision making when attending elderly fall patients/normal care | Opt out |
| Steg *et al.* (2013) | France, Netherlands, Denmark, Poland, Italy, Austria, Germany, Slovenia, UK | STEMI | Bivalirudin/Normal care | Initial verbal consent with full informed consent. |
| Stub *et al*. (2012) | Australia | STEMI | Oxygen/normal care | Delayed consent or relative proxy consent where the patient does not regain capacity |
| ten Berg *et al*. (2010) | Netherlands, Germany | STEMI | Tirofiban/normal care | Full informed consent in the ambulance |
| Thompson *et al*. (2008) | Canada | Acute respiratory failure | CPAP/normal care | Brief verbal consent by patient or relative at scene. Delayed consent from patient or relative proxy consent where the patient does not regain capacity |
| van’t Hof *et al*. (2008) | Netherlands, Germany, Belgium | STEMI | Tirofiban/placebo | Full informed consent in the ambulance |
| Vincente *et al*. (2014) | Sweden | Elderly patients | Ambulance based triage/normal care | Full informed consent or relative proxy consent where the patient lacked capacity |
| Walter *et al*. (2012) | Germany | Stroke | Mobile stroke unit pathway/normal care | Full informed consent either in the mobile stroke unit or the hospital for the control group. |
| Wendt *et al*. (2015) | Germany | Stroke | Mobile stroke unit pathway/normal care | Follow up data collected from patients that had previously suppled full informed consent |

**Reference List**

Ankolekar, S., Fuller, M., Cross, I., Renton, C., Cox, P., Sprigg, N., Siriwardena, A.N. and Bath, P.M. (2013) “Feasibility of an ambulance-based stroke trial, and safety of glyceryl trinitrate in ultra-acute stroke: The rapid intervention with glyceryl trinitrate in hypertensive stroke trial.” *Stroke (00392499),* **44(11)**, 3120-3128.

Ankolekar, S., Sare, G., Geeganage, C., Fuller, M., Stokes, L., Sprigg, N., Parry, R., Siriwardena, A.N. and Bath, P.M.W. (2012) “Determining the feasibility of ambulance-based randomised controlled trials in patients with ultra-acute stroke: Study protocol for the "rapid intervention with GTN in hypertensive stroke trial" (RIGHT, ISRCTN66434824)” *Stroke Research & Treatment,* 1-10.

Arendts, G., Sim, M., Johnston, S. and Brightwell, R. (2011) “ParaMED home: A protocol for a randomised controlled trial of paramedic assessment and referral to access medical care at home.” *BMC Emergency Medicine,* **11(1)** 7-10.

Baker, P.W., Conway, J., Cotton, C., Ashby, D.T., Smyth, J., Woodman, R.J. and Grantham, H. (2008) “Defibrillation or cardiopulmonary resuscitation first for patients with out-of-hospital cardiac arrests found by paramedics to be in ventricular fibrillation? A randomised control trial.” *Resuscitation,* **79(3)** 424-43

Barker, R., Lang, T., Steinlechner, B., Mora, B., Heigel, P., Gauss, N., Zimpfer, M. and Kober, A. (2006) “Transcutaneous electrical nerve stimulation as prehospital emergency interventional care: Treating acute pelvic pain in young women.” *Neuromodulation*, **9(2)** 136-142.

Benger, J., Coates, D., Davies, S., Greenwood, R., Nolan, J., Rhys, M., Thomas, M. and Voss, S. (2016) “Randomised comparison of the effectiveness of the laryngeal mask airway supreme, i-gel and current practice in the initial airway management of out of hospital cardiac arrest: A feasibility study.” *BJA: The British Journal of Anaesthesia,* **116(2)** 262-268

Bernard, S.A., Smith, K., Cameron, P., Masci, K., McD. Taylor, D., Cooper, D.J., Kelly, A. and Silvester, W. (2012) “Induction of resuscitation prehospital therapeutic hypothermia after from nonventricular fibrillation cardiac arrest.” *Critical Care Medicine,* **40(3)** 747-753

Brasel, K.J., Bulger, E., Cook, A.J., Morrison, L.J., Newgard, C.D., Tisherman, S.A., Kerby, J.D., Coimbra, R., Hata, J.S. and Hoyt, D.B. (2008) “Hypertonic resuscitation: Design and implementation of a prehospital intervention trial.” *Journal of the American College of Surgeons,* **206(2)** 220-232

Breil, M., Krep, H., Heister, U., Bartsch, A., Bender, R., Schaefers, B., Hoeft, A. and Fischer, M. (2012) “Randomised study of hypertonic saline infusion during resuscitation from out-of-hospital cardiac arrest.” *Resuscitation,* **83(3)** 347-352

Brown, S.P., Wang, H., Aufderheide, T.P., Vaillancourt, C., Schmicker, R.H., Cheskes, S., Straight, R., Kudenchuk, P., Morrison, L., Colella, M.R., Condle, J., Gamez, G., Hostler, D., Kayea, T., Ragsdale, S., Stephens, S. and Nichol, G. (2015) “A randomized trial of continuous versus interrupted chest compressions in out-of-hospital cardiac arrest: Rationale for and design of the resuscitation outcomes consortium continuous chest compressions trial.” *American Heart Journal,* **169(3)** 334-341.

Dixon, S., Mason, S., Knowles, E., Colwell, B., Wardrope, J., Snooks, H., Gorringe, R., Perrin, J. and Nicholl, J. (2009) “Is it cost effective to introduce paramedic practitioners for older people to the ambulance service? results of a cluster randomised controlled trial.” *Emergency Medicine Journal: EMJ,* **26(6)** 446-451

Ducasse, J., Siksik, G., Durand-Bechu, M., Couarraze, S., Valle, B., Lecoules, N., Marco, P., Lacombe, T. and Bounes, V. (2013) “Nitrous Oxide for Early Analgesia in the Emergency Setting: A Randomized, Doubleblind Multicenter Prehospital Trial.” *Academic Emergency Medicine* **20(2)** 178-184

Ebinger, M., Winter, B., Wendt, M., Weber, J.E., Waldschmidt, C., Rozanski, M., Kunz, A., Koch, P., Kellner, P.A., Gierhake, D., Villringer, K., Fiebach, J.B., Grittner, U., Hartmann, A., Mackert, B., Endres, M. and Audebert, H.J. (2014) “Effect of the use of ambulance-based thrombolysis on time to thrombolysis in acute ischemic stroke.” *JAMA: Journal of the American Medical Association,* **311(16)** 1622-1631

Ezekowitz, J.A., Welsh, R.C., Gubbels, C., Brass, N., Chan, M., Keeble, W., Khadour, F., Koshy, T.L., Knapp, D., Sharma, S., Sookram, S., Tymchak, W., Weiss, D., Westerhout, C.M. and Armstrong, P.W. (2014) “Providing rapid out of hospital acute cardiovascular treatment 3 (PROACT-3).” *Canadian Journal of Cardiology,* **30(10)** 1208-1215.

Frascone, R.J., Russi, C., Lick, C., Conterato, M., Wewerka, S.S., Griffith, K.R., Myers, L., Conners, J. and Salzman, J.G. (2011) “Comparison of prehospital insertion success rates and time to insertion between standard endotracheal intubation and a supraglottic airway.” *Resuscitation,* **82(12)** 1529-1536.

Garner, A. A., Mann, K. P., Fearnside, M., Poynter, E. and Gebski, V. (2015) “The head injury retrieval trial (HIRT): A single-centre randomised controlled trial of physician prehospital management of severe blunt head injury compared with management by paramedics only.” *Emergency Medicine Journal,* **32(11)** 869-875

Hassan, T.B., Jagger, C. and Barnett, D.B. (2002) “A randomised trial to investigate the efficacy of magnesium sulphate for refractory ventricular fibrillation.” *Emergency Medicine Journal,* **19(1)** 57-62

Heestermans, T., Suryapranata, H., ten Berg, J.M., Mosterd, A., Gosselink, A.T.M., Kochman, W., Dill, T., van Houwelingen, G., Kolkman, E., van Werkum, J.W., Zijlstra, F., Hamm, C. and van 't Hof, A.W.J. (2011) “Facilitated reperfusion with prehospital glycoprotein IIb/IIIa inhibition: Predictors of complete ST-segment resolution before primary percutaneous coronary intervention in the on-TIME 2 trial: Correlates of reperfusion before primary PCI.” *Journal of Electrocardiology,* **44(1)** 42-48.

Heestermans, T., van 't Hof, A.W.J., ten Berg, J.M., van Werkum, J.W., Boersma, E., Mosterd, A., Stella, P.R., van Zoelen, A.B., Gosselink, A.T.M., Kochman, W., Dill, T., Koopmans, P.C., van Houwelingen, G., Zijlstra, F. and Hamm, C. (2010) “The golden hour of prehospital reperfusion with triple antiplatelet therapy: A sub-analysis from the ongoing tirofiban in myocardial evaluation 2 (on-TIME 2) trial early initiation of triple antiplatelet therapy.” *American Heart Journal,* **160(6)** 1079-1084

Jabre, P., Galinski, M., Ricard-Hibon, A., Devaud, M.L., Ruscev, M., Kulstad, E., Vicaut, E., Adnet, F., Margenet, A., Marty, J. and Combes, X. (2011) “Out-of-hospital tracheal intubation with single-use versus reusable metal laryngoscope blades: A multicenter randomized controlled trial.” *Annals of Emergency Medicine,* **57(3)** 225-231.

Jacobs, I.G., Finn, J.C., Jelinek, G.A., Oxer, H.F. and Thompson, P.L. (2011) “Effect of adrenaline on survival in out-of-hospital cardiac arrest: A randomised double-blind placebo-controlled trial.” *Resuscitation,* **82(9)** 1138-1143

Jacoby, J., Heller, M., Nicholas, J., Patel, N., Cesta, M., Smith, G., Jacob, S. and Reed, J. (2006) “Etomidate versus midazolam for out-of-hospital intubation: A prospective, randomized trial.” *Annals of Emergency Medicine,* **47(6)** 525-530.

Jennings, P.A., Cameron, P., Bernard, S., Walker, T., Jolley, D., Fitzgerald, M. and Masci, K. (2012) “Morphine and ketamine is superior to morphine alone for out-of-hospital trauma analgesia: A randomized controlled trial.” *Annals of Emergency Medicine,* **59(6)** 497-503.

Jennings, P.A., Cameron, P., Bernard, S., Walker, T., Jolley, D., Fitzgerald, M. and Masci, K. (2014) “Long-term pain prevalence and health-related quality of life outcomes for patients enrolled in a ketamine versus morphine for prehospital traumatic pain randomised controlled trial.” *Emergency Medicine Journal,* **31(10)** 840-843

Kämäräinen, A., Virkkunen, I., Tenhunen, J., Yli-Hankala, A. and Silfvast, T. (2009) “Prehospital therapeutic hypothermia for comatose survivors of cardiac arrest: A randomized controlled trial.” *Acta Anaesthesiologica Scandinavica,* **53(7)** 900-907

Kim, F., Nichol, G., MD, Maynard, C., Hallstrom, A., Kudenchuk, P.J., Rea, T., Copass, M.K., MD; Carlbom, D., Deem, S., Longstreth Jr, W.T., Olsufka, M., Cobb, L.A. (2014) “Effect of Prehospital Induction of Mild Hypothermia on Survival and Neurological Status Among Adults With Cardiac Arrest: A randomized controlled trial.” *JAMA: Journal of the American Medical Association,* **311(1)** 45-52

Kober, A., Fleischackl, R., Scheck, T., Lieba, F., Strasser, H., Friedmann, A. and Sessler, D.I. (2002) “A randomized controlled trial of oxygen for reducing nausea and vomiting during emergency transport of patients older than 60 years with minor trauma.” *Mayo Clinic Proceedings,* **77(1)** 35-38

Kovoor, P., Love, A., Hall, J., Kruit, R., Sadick, N., Ho, D., Adelstein, B. and Ross, D.L. (2005) “Randomized double-blind trial of sotalol versus lignocaine in out-of-hospital refractory cardiac arrest due to ventricular tachyarrhythmia.” *Internal Medicine Journal,* **35(9)** 518-525

Larsson, G., Strömberg, R.U., Rogmark, C. and Nilsdotter, A. (2016) “Prehospital fast track care for patients with hip fracture: Impact on time to surgery, hospital stay, postoperative complications and mortality A randomized, controlled trial.” *Injury,* **47(4)** 881-886

Lerner, E.B., Persse, D., Souders, C.M., Sterz, F., Malzer, R., Lozano Jr., M., Westfall, M., Brouwer, M.A., van Grunsven, P.M., Whitehead, A., Olsen, J., Herken, U.R. and Wik, L. (2011) “Design of the circulation improving resuscitation care (CIRC) trial: A new state of the art design for out-of-hospital cardiac arrest research.” *Resuscitation,* **82(3)** 294-299

McRae, P.J., Bendall, J.C., Madigan, V. and Middleton, P.M. (2015) “Paramedic-performed fascia iliaca compartment block for femoral fractures: A controlled trial.” *Journal of Emergency Medicine (0736-4679),* **48(5)** 581-589.

Middleton, P.M., Simpson, P.M., Thomas, R.E. and Bendall, J.C. (2014) “Higher insertion success with the i-gel® supraglottic airway in out-of-hospital cardiac arrest: A randomised controlled trial.” *Resuscitation,* **85(7)** 893-897.

Montalescot, G., Lassen, J.F., Hamm, C.W., Lapostolle, F., Silvain, J., Ten Berg, J.,M., Cantor, W.J., Goodman, S.G., Licour, M., Tsatsaris, A. and Van't Hof, A.,W. (2013) “Ambulance or in-catheterization laboratory administration of ticagrelor for primary percutaneous coronary intervention for ST-segment elevation myocardial infarction: Rationale and design of the randomized, double-blind administration of ticagrelor in the cath Lab or in the Ambulance for New ST elevation myocardial Infarction to open the Coronary artery (ATLANTIC) study.” *American Heart Journal,* **165(4)** 515-522

Moore, C. and Woollard, M. (2005) “Dextrose 10% or 50% in the treatment of hypoglycaemia out of hospital? A randomised controlled trial.” *Emergency Medicine Journal,* **22(7)** 512-515

Morrison, L.J., Dorian, P., Long, J., Vermeulen, M., Schwartz, B., Sawadsky, B., Frank, J., Cameron, B., Burgess, R., Shield, J., Bagley, P., Mausz, V., Brewer, J.E. and Lerman, B.B. (2005) “Out-of-hospital cardiac arrest rectilinear biphasic to monophasic damped sine defibrillation waveforms with advanced life support intervention trial (ORBIT).” *Resuscitation,* **66(2)** 149-157

Morrison, L.J., Long, J., Vermeulen, M., Schwartz, B., Sawadsky, B., Frank, J., Cameron, B., Burgess, R., Shield, J., Bagley, P., Mausz, V., Brewer, J.E. and Dorian, P. (2008) “A randomized controlled feasibility trial comparing safety and effectiveness of prehospital pacing versus conventional treatment: ‘PrePACE’” *Resuscitation,* **76(3)** 341-349

Olasveengen, T.M., Wik, L., Sunde, K. and Steen, P.A. (2012) “Outcome when adrenaline (epinephrine) was actually given vs. not given – post hoc analysis of a randomized clinical trial.” *Resuscitation,* **83(3)** 327-332

Paradis, N.A., Young, G., Lemeshow, S., Brewer, J.E. and Halperin, H.R. (2010) “Inhomogeneity and temporal effects in AutoPulse Assisted Prehospital International Resuscitation—an exception from consent trial terminated early.” *American Journal of Emergency Medicine* **28** 391–398

Perkins, G.D., Lall, R., Quinn, T., Deakin, C.D., Cooke, M.W., Horton, J., Lamb, S.E., Slowther, A., Woollard, M., Carson, A., Smyth, M., Whitfield, R., Williams, A., Pocock, H., Black, J.J.M., Wright, J., Han, K. and Gates, S. (2015) “Mechanical versus manual chest compression for out-of-hospital cardiac arrest (PARAMEDIC): A pragmatic, cluster randomised controlled trial.” *The Lancet,* **385(9972)** 947-955

Perkins, G.D., Woollard, M., Cooke, M.W., Deakin, C., Horton, J., Lall, R., Lamb, S.E., McCabe, C., Quinn, T., Slowther, A. and Gates, S (2010) “Prehospital randomised assessment of a mechanical compression device in cardiac arrest (PaRAMeDIC) trial protocol.” *Scandinavian Journal of Trauma, Resuscitation and Emergency Medicine,* **18** 58-58

Rubertsson, S., Lindgren, E., Smekal, D., Östlund, O., Silfverstolpe, J., Lichtveld, R.A., Boomars, R., Ahlstedt, B., Skoog, G., Kastberg, R., Halliwell, D., Box, M., Herlitz, J. and Karlsten, R. (2014) “Mechanical chest compressions and simultaneous defibrillation vs conventional cardiopulmonary resuscitation in out-of-hospital cardiac arrest:  The LINC randomized trial.” *The Journal of the American Medical Association,* **311(1)** 53-61

Shaw, L., Price, C., McLure, S., Howel, D., McColl, E. and Ford, G.A. (2011) “Paramedic initiated lisinopril for acute stroke treatment (PIL-FAST): Study protocol for a pilot randomised controlled trial.” *Trials,* **12** 152-152.

Shaw, L., Price, C., McLure, S., Howel, D., McColl, E., Younger, P. and Ford, G.A. (2014) “Paramedic initiated lisinopril for acute stroke treatment (PIL-FAST): Results from the pilot randomised controlled trial.” *Emergency Medicine Journal,* **31(12)** 994-999

Snooks, H., Anthony, R., Chatters, R., Cheung, W., Dale, J., Donohoe, R., Gaze, S., Halter, M., Koniotou, M., Logan, P., Lyons, R., Mason, S., Nicholl, J., Phillips, C., Phillips, J., Russell, I., Siriwardena, A.N., Wani, M., Watkins, A., Whitfield, R. and Wilson, L. (2012) “Support and assessment for fall emergency referrals (SAFER 2) research protocol: Cluster randomised trial of the clinical and cost effectiveness of new protocols for emergency ambulance paramedics to assess and refer to appropriate community-based care.” *BMJ Open,* **2(6)**.

Snooks, H., Cheung, W., Close, J., Dale, J., Gaze, S., Humphreys, I., Lyons, R., Mason, S., Merali, Y., Peconi, J., Phillips, C., Phillips, J., Roberts, S., Russell, I., Sánchez, A., Wani, M., Wells, B. and Whitfield, R. (2010) “Support and assessment for fall emergency referrals (SAFER 1) trial protocol. computerised on-scene decision support for emergency ambulance staff to assess and plan care for older people who have fallen: Evaluation of costs and benefits using a pragmatic cluster randomised trial.” *BMC Emergency Medicine,* **10** 2-2

Steg, P.G., van ‘t Hof, A., Clemmensen, P., Lapostolle, F., Dudek, D., Hamon, M., Cavallini, C., Gordini, G., Huber, K., Coste, P., Thicoipe, M., Nibbe, L., Steinmetz, J., Ten Berg, J., Eggink, G.J., Zeymer, U., Campo dell'Orto, M., Kanic, V., Deliargyris, E.N., Day, J., Schuette, D., Hamm, C.W. and Goldstein, P. (2013) “Design and methods of european ambulance acute coronary syndrome angiography trial (EUROMAX): An international randomized open-label ambulance trial of bivalirudin versus standard-of-care anticoagulation in patients with acute ST-segment-elevation myocardial infarction transferred for primary percutaneous coronary intervention.” *American Heart Journal,* **166(6)** 960-967

Stub, D., Smith, K., Bernard, S., Bray, J.E., Stephenson, M., Cameron, P., Meredith, I. and Kaye, D.M. (2012) “A randomized controlled trial of oxygen therapy in acute myocardial infarction air verses oxygen in myocarDial infarction study (AVOID study).” *American Heart Journal,* **163(3)** 339-345.

Stub, D., Smith, K., Bernard, S., Nehme, Z., Stephenson, M., Bray, J.E., Cameron, P., Barger, B., Ellims, A.H., Taylor, A.J., Meredith, I.T. and Kaye, D.M. (2015) “Air versus oxygen in ST-segment-elevation myocardial infarction.” *Circulation,* **131(24)** 2143-2150

ten Berg, J.M., van 't Hof, A.W.J., Dill, T., Heestermans, T., van Werkum, J.W., Mosterd, A., van Houwelingen, G., Koopmans, P.C., Stella, P.R., Boersma, E. and Hamm, C. (2010) “Effect of early, pre-hospital initiation of high bolus dose tirofiban in patients with ST-segment elevation myocardial infarction on short- and long-term clinical outcome.” *Journal of the American College of Cardiology,* **55(22)** 2446-2455

Thompson, J., Petrie, D.A., Ackroyd-Stolarz, S. and Bardua, D.J. (2008) “Out-of-hospital continuous positive airway pressure ventilation versus usual care in acute respiratory failure: A randomized controlled trial.” *Annals of Emergency Medicine,* **52(3)** 232-241

van't Hof, A.W., ten Berg, J., Heestermans, T., Dill, T., Funck, R.C., van Werkum, W., Dambrink, J.E., Suryapranata, H., van Houwelingen, G., Ottervanger, J.P., Stella, P., Giannitsis, E. and Hamm, C. (2008) “Prehospital initiation of tirofiban in patients with ST-elevation myocardial infarction undergoing primary angioplasty (on-TIME 2): A multicentre, double-blind, randomised controlled trial.” *The Lancet,* **372(9638)** 537-546.

Vicente, V., Svensson, L., Wireklint Sundström, B., Sjöstrand, F. and Castren, M. (2014) “Randomized controlled trial of a prehospital decision system by emergency medical services to ensure optimal treatment for older adults in Sweden.” *Journal of the American Geriatrics Society,* **62(7)** 1281-1287

Walter, S., Kostopoulos, P., Haass, A., Keller, I., Lesmeister, M., Schlechtriemen, T., Roth, C., Papanagiotou, P., Grunwald, I., Schumacher, H., Helwig, S., Viera, J., Körner, H., Alexandrou, M., Yilmaz, U., Ziegler, K., Schmidt, K., Dabew, R., Kubulus, D., Liu, Y., Volk, T., Kronfeld, K., Ruckes, C., Bertsch, T., Reith, W. and Fassbender, K. (2012) “Diagnosis and treatment of patients with stroke in a mobile stroke unit versus in hospital: A randomised controlled trial.” *The Lancet Neurology,* **11(5)** 397-404

Wendt, M., Ebinger, M., Kunz, A., Rozanski, M., Waldschmidt, C., Weber, J.E., Winter, B., Koch, P.M., Freitag, E., Reich, J., Schremmer, D. and Audebert, H.J. (2015) “Improved prehospital triage of patients with stroke in a specialized stroke ambulance: Results of the pre-hospital acute neurological therapy and optimization of medical care in stroke study.” *Stroke (00392499),* **46(3)** 740-745
